# Histological subtypes of ovarian cancer associated with parity and breastfeeding in the prospective Million Women Study

**DOI:** 10.1002/ijc.31063

**Published:** 2017-10-12

**Authors:** Kezia Gaitskell, Jane Green, Kirstin Pirie, Isobel Barnes, Carol Hermon, Gillian K Reeves, Valerie Beral, Million Women Study Collaborators

**Affiliations:** ^1^ Cancer Epidemiology Unit, Nuffield Department of Population Health University of Oxford, Richard Doll Building, Roosevelt Drive Oxford OX3 7LF United Kingdom

**Keywords:** parity, breastfeeding, reproductive factors, ovarian cancer, histological type

## Abstract

Ovarian cancer risk is known to be reduced amongst women who have had children, but reported associations with breastfeeding are varied. Few studies have had sufficient power to explore reliably these associations by tumour histotype. In a prospective study of 1.1 million UK women, 8719 developed ovarian cancer during follow‐up. Cox regression yielded adjusted relative risks (RRs) overall and by tumour histotype amongst women with different childbearing patterns. Nulliparous women had a 24% greater ovarian cancer risk than women with one child, with significant heterogeneity by histotype (*p* = 0.01). There was no significant increase in serous tumours, a modest increase in mucinous tumours, but a substantial increase in endometrioid (RR = 1.49, 95% CI: 1.18‐1.89) and clear‐cell tumours (RR = 1.68, 1.29‐2.20). Among parous women, each additional birth was associated with an overall 6% reduction in ovarian cancer risk; this association also varied by histotype (p = 0.0006), with the largest reduction in risk for clear‐cell tumours (RR per birth = 0.75, 0.65‐0.85, *p* < 0.001) and weak, if any, effect for endometrioid, high‐grade serous, or mucinous tumours. We found little association with age at first or last birth. There was about a 10% risk reduction per 12‐months breastfeeding (RR = 0.89, 0.84‐0.94, *p* < 0.001), with no significant heterogeneity by histotype, but statistical power was limited. In this large prospective study, ovarian cancer risk associated with parity varied substantially by tumour histotype. Nulliparity was associated with a substantially greater overall risk than expected from the effect of a single birth, especially for clear cell and endometrioid tumours, perhaps suggesting that infertility is associated with these histotypes.

## Abstract 2

**What's new?**

Evidence suggests that women who bear children are at decreased risk of ovarian cancer. Whether breastfeeding contributes to this reduction in risk remains unclear. In this prospective study, each birth in parous women reduced overall ovarian cancer risk by 6%, while nulliparity was associated with a 24% increase in risk compared to women with one child. Among parous women, risk reduction varied by tumour type, with the greatest reduction observed for clear cell tumours and almost no reduction detected for serous tumours. Ovarian cancer risk was additionally reduced by 10% for every 12 months of breastfeeding.

**Abbreviations**
95% CI: 95% Confidence Interval
BMI: Body Mass Index
ICD‐10: International Classification of Diseases, version 10
ICD‐O: International Classification of Diseases for Oncology
MWS: the Million Women Study
NHS: the National Health Service
RR: Relative Risk
SD: Standard Deviation
WHO: World Health Organization

Many epidemiological studies have reported a reduced risk of ovarian cancer amongst women who have had children, finding decreasing risk with increasing number of births.^1^, ^2^, ^3^, ^4^, ^5^, ^6^, ^7^, ^8^, ^9^, ^10^, ^11^, ^12^, ^13^, ^14^, ^15^, ^16^, ^17^ Infertility has been associated with an increased risk of ovarian cancer,^18^, ^19^ but few studies have examined the effects in nulliparous women, many of whom may be infertile.^20^, ^21^ Long durations of breastfeeding have also been associated with a reduced risk of ovarian cancer,^22^ although there is considerable heterogeneity in the findings, not all of which included adequate adjustment for the number of children women have had, which is highly correlated with total duration of breastfeeding.

The majority of cases of ovarian cancer are epithelial tumours, of which the most common histological type is high‐grade serous carcinoma (about 60%); the next most common histotypes are endometrioid, clear cell, mucinous, and low‐grade serous carcinomas, each of which accounts for <20% of the total.^23^ Recent evidence from histopathological and genetic studies suggests that the different histotypes of ovarian cancer may have distinct, possibly extra‐ovarian, origins. For example, it is suggested that many high‐grade serous tumours may arise from precursor lesions within the fallopian tubes, while some endometrioid and clear cell tumours may develop from endometriosis; the origins of mucinous tumours are uncertain.^24^ Many factors, including use of oral contraceptives,^25^ use of menopausal hormones,^26^ and smoking^27^ show variation in risk by ovarian tumour histotype.

Some^2^, ^6^^,^ 13, 17 but not all^5^, ^7^^,^ 15 epidemiological studies have reported that some associations between childbearing patterns and ovarian cancer varied by histological type, although few formally tested for heterogeneity and there is little consistency in the findings across studies. No single previous study with information on potential confounding factors (such as use of the oral contraceptive pill) included >2,000 cases of ovarian cancer, too few to examine reliably for heterogeneity by histotype.

A pooled analysis of studies with retrospective ascertainment of childbearing history included 5,566 cases of ovarian cancer, and limited results on parity and risk of ovarian cancer histotypes were reported just as part of an analysis of a genetic risk score in relation to environmental factors.^28^ A pooled analysis of 21 prospective studies from the Ovarian Cancer Cohort Consortium included 5,584 cases of invasive epithelial ovarian cancer, and found strong evidence of an association between parity and ovarian cancer risk, with heterogeneity by histotype, but no significant association with breastfeeding, either overall or by histotype.^29^

Large numbers are needed to examine reliably the association between reproductive factors (parity and breastfeeding) and risk by ovarian cancer histotype, and we report here findings from a prospective study of one million UK women with almost 9,000 incident cases of ovarian cancer.

## Material and Methods

### Study design, data collection and follow‐up

The Million Women Study is a cohort of 1.3 million UK women, aged 56 (standard deviation (SD) 5) at the time of recruitment (1996–2001). Participants were invited to take part in the study via letters sent with invitations to attend mammographic screening for breast cancer. The women completed questionnaires at recruitment, including information on reproductive, sociodemographic, and health factors. The study methods have been described in detail elsewhere,^30^ and study questionnaires can be viewed at http://www.millionwomenstudy.org. Information on data access for the Million Women Study is available at www.millionwomenstudy.org/data_access/. Follow‐up was via record linkage to the National Health Service (NHS) Central Register (based on unique NHS number and other personal identifiers): all participants were ‘flagged’, so that the study investigators were routinely notified of cancer registrations or deaths. Cancer diagnoses were coded by the NHS Central Register using the 10th revision of the WHO International Classification of Diseases (ICD‐10),^31^ with morphology codes from the 2nd and 3rd edition of the International Classification of Diseases for Oncology (ICD‐O).^32^, ^33^

All participants gave written consent to follow‐up at recruitment. The study received ethical approval from the Oxford and Anglia Multi‐Centre Research Ethics Committee (MREC 97/01).

### Exposure variables

Information on reproductive factors was taken from the recruitment questionnaire. Parity was defined by the number of full‐term pregnancies, derived from the response to the question: ‘How many children have you had? (please include stillbirths; it is not necessary to include miscarriages)’ and the dates of birth for each child. The latter information, as recorded by the women, was also used for analyses of the woman's age at first or last birth.

Total duration of breastfeeding was calculated as the sum of the duration of breastfeeding each child, as reported in response to the question: ‘for how many months did you breastfeed each child, if at all?’ Average duration of breastfeeding per child breastfed was calculated as the sum of duration of breastfeeding each child, divided by the number of children breastfed.

### Outcome

The outcome of interest was ovarian cancer (ICD‐10 C56). For analyses of histotype, the outcome was split into the four most common specific histological groups: serous (ICD‐O codes 8441–8442, 8451, 8460–8463, 9014), mucinous (ICD‐O codes 8470–8490), endometrioid (ICD‐O codes 8380–8381, 8560, 8570), and clear cell (ICD‐O codes 8310, 8313); the remainder of cases were classified as other/unspecified.

For some analyses, serous and mucinous tumours were further subdivided, as previously described.^34^ Serous tumours were divided into low‐grade tumours (defined here as borderline serous tumours (ICD‐O codes 8442, 8451, 8462, 8463) or grade 1 serous carcinomas), and high‐grade serous carcinomas (defined here as grade ≥2 serous carcinomas). Mucinous tumours were split into mucinous borderline tumours (ICD‐O codes 8472–8473) and mucinous carcinomas (ICD‐O codes 8470–8471 and 8480–8490).

### Statistical analyses

Women were excluded if they had been diagnosed with any invasive cancer (other than non‐melanoma skin cancer, ICD‐10 C44) prior to recruitment (*n* = 44,775); if they reported a previous bilateral oophorectomy (or were unsure, or the information was missing) (*n* = 170,772); or if they had missing information on parity (*n* = 3,883). The remaining women (*N* = 1,144,762) contributed person‐years from the date of recruitment into the study until the date of registration for ovarian cancer, the date of death, or last date of follow‐up (31 December 2014)—whichever was soonest. Women were censored at diagnosis of any cancer (other than non‐melanoma skin cancer). About 1% of participants were lost to follow‐up by the end of 2014 and such women are censored at the date when they were lost, contributing person‐years until then.

We used Cox proportional hazards models to estimate hazard ratios (referred to as relative risks [RRs]) of ovarian cancer according to parity, age at first or last birth, and breastfeeding. Attained age was the underlying time variable. There was no evidence of significant violation of the proportional hazards assumption, as assessed by graphical methods and tests based on Schoenfeld residuals.

In analyses of parity, we first estimated the RR for parous versus nulliparous women. We then estimated the RR for nulliparous vs. para‐1 women and the RR per birth in parous women by simultaneously modelling parity as a binary variable indicating nulliparous women and as a linear trend in parous women (through 1, 2, 3, 4, 5.5 births; 5.5 is the mean number of births in women who had 5+ births). Heterogeneity of these two RRs was tested using a contrast test. In categorical analyses of parity and ovarian cancer risk by histological type among parous women, parity was categorised into 3 groups (1, 2, 3+ births) due to the small number of cases of less common subtypes with high parity.

Analyses of the timings of births were restricted to parous women with complete information on age at first and last births, and were stratified by parity (1, 2, 3+). Both age at first birth, and age at last birth, were treated as ordered categorical variables (age at first birth: <20, 20–24, 25+; age at last birth: <25, 25–29, 30+). Tests for trend used the categorical variables coded with the median age in each age‐group (age at first birth: 18, 22, 27; age at last birth: 23, 27, 33). Separate models were run for age at first birth and age at last birth.

Analyses of breastfeeding used the total duration of breastfeeding (summed over all births), or the average duration of breastfeeding per child breastfed, as ordered categorical variables (none, ≤1 month, >1 and <6 months, ≥6 and <12 months, 12+ months). Tests for trend used the categorical variable coded by the median duration of breastfeeding in each group (total duration: 0, 1, 3, 8, 17 months; average duration per child breastfed: 0, 0.67, 3, 7, 13.5 months). For analyses of breastfeeding, women were excluded if they had missing information on the duration of breastfeeding (information was missing for approximately 20% of women, as this question was not included in the earliest batch of questionnaires). All breastfeeding analyses were restricted to parous women, and stratified by parity (1, 2, 3, 4, 5+).

All analyses were stratified by geographical region (10 regions corresponding to the areas covered by the cancer registries), and adjusted for use of the oral contraceptive pill (never, ever), tubal ligation (no, yes), family history of breast cancer (no, yes), hysterectomy (no, yes), use of menopausal hormones (never, ever), body mass index (BMI) (<25 kg/m^2^, 25–29 kg/m^2^, 30+ kg/m^2^), smoking history (never, past, current), and quintiles of socioeconomic status based on the Townsend deprivation index.^35^ For adjustment variables, missing values were assigned to a separate category. All information on adjustment variables was taken from the recruitment questionnaire. Exposure information was either missing or reported as unknown for <5% of women for all potential confounders.

Other factors (including alcohol consumption, physical activity, and age at menarche) were explored as potential confounders, but were not included in the final model as their inclusion made no appreciable difference to the main estimate of effect.

Tests of heterogeneity in the relationship between ovarian cancer and parity or breastfeeding by histotype were performed using a competing risks approach.^36^

For analyses where comparisons were made between more than two exposure categories, relative risks are presented in figures with group‐specific confidence intervals (95% g‐s CI) for the log risk in each group (allowing comparisons to be made between any two categories, even if neither is the reference group).^37^, ^38^ Conventional 95% confidence intervals are given in the text. All analyses were performed in Stata‐14.^39^ Figures were drawn in R^40^ using either ‘ggplot2’^41^ or the in‐house package ‘Jasper’.^42^

## Results

A total of 1,144,762 women were included in this analysis, with a mean age at recruitment of 56.1 years (SD 4.8). Overall, 89% of participants reported at least one full‐term pregnancy. Table 1 shows the characteristics of the study population, by parity. Compared to nulliparous women, parous women were more likely to have used the oral contraceptive pill and menopausal hormones, to have undergone tubal ligation and hysterectomy, and to be current smokers.

**Table 1:** Characteristics of the study population at recruitment, and details of follow‐up, according to parity

	Parity		
Characteristics	Nulliparous	Parous	All women
Number of women	123,927	1,020,835	1,144,762
Mean (SD) age at recruitment (years)	56.1 (5.0)	56.1 (4.8)	56.1 (4.8)
Socioeconomic status, lower third, % (*n*)	30.7 (37,774)	32.3 (327,624)	32.2 (365,398)
Mean (SD) age at menarche	12.9 (1.6)	13.0 (1.6)	13.0 (1.6)
Ever use of oral contraceptive pill, % (*n*)	45.6 (56,054)	61.6 (624,076)	59.9 (680,130)
Tubal ligation, % (*n*)	5.0 (6,149)	23.8 (239,239)	21.7 (245,388)
Ever use of menopausal hormones, % (*n*)	42.0 (51,683)	47.5 (479,976)	46.9 (531,659)
Hysterectomy, % (*n*)	10.3 (12,796)	16.9 (172,168)	16.2 (184,964)
Mean (SD) age at natural menopause	48.7 (4.4)	49.2 (4.2)	49.1 (4.2)
Family history of breast cancer, % (*n*)	10.0 (11,615)	9.8 (94,349)	9.9 (105,964)
Mean (SD) body mass index (kg/m^2^)	25.7 (4.8)	26.2 (4.6)	26.2 (4.7)
Current smoker, % (*n*)	17.1 (20,130)	20.5 (197,543)	20.2 (217,673)
Strenuous exercise ≥once/week, % (*n*)	39.9 (47,959)	39.2 (385,852)	39.3 (433,811)
Alcohol intake, ≥7 units/week, % (*n*)	27.2 (33,473)	23.6 (238,840)	24.0 (272,313)
**Follow‐up for cancer incidence**			
Woman‐years of follow‐up (100,000s)	17.8	149.5	167.3
Mean follow‐up time per woman (SD)	14.3 (4.0)	14.6 (3.6)	14.6 (3.7)
Number of incident cancers	1,266	7,453	8,719
Mean (SD) age at ovarian cancer diagnosis	65.2 (6.7)	65.7 (6.5)	65.6 (6.5)

Notes: Means and percentages are calculated excluding missing values for the variable of interest.

*n*: number of women; SD: standard deviation.

During 16.7 million person‐years of follow‐up (an average of 14.6 [SD 3.7] years per woman), 8719 incident ovarian cancers were reported, with a mean age at diagnosis of 65.6 years (SD 6.5). Of the 5848 epithelial cancers with the four most common tumour types, 67% were serous (*n* = 3916), 15% mucinous (*n* = 896), 11% endometrioid (*n* = 622) and 7% clear cell (*n* = 414). Of the remaining cases (*n* = 2871), the majority were recorded as unspecified carcinoma or adenocarcinoma.

### Parity and risk of ovarian cancer

Overall, parous women had an estimated 26% lower risk of ovarian cancer than nulliparous women (RR = 0.74, 95% CI: 0.70–0.79), after adjustment for age, region, socioeconomic status, tubal ligation, family history of breast cancer, hysterectomy, BMI, smoking, and use of contraceptive or menopausal hormones.

**Figure 1:**
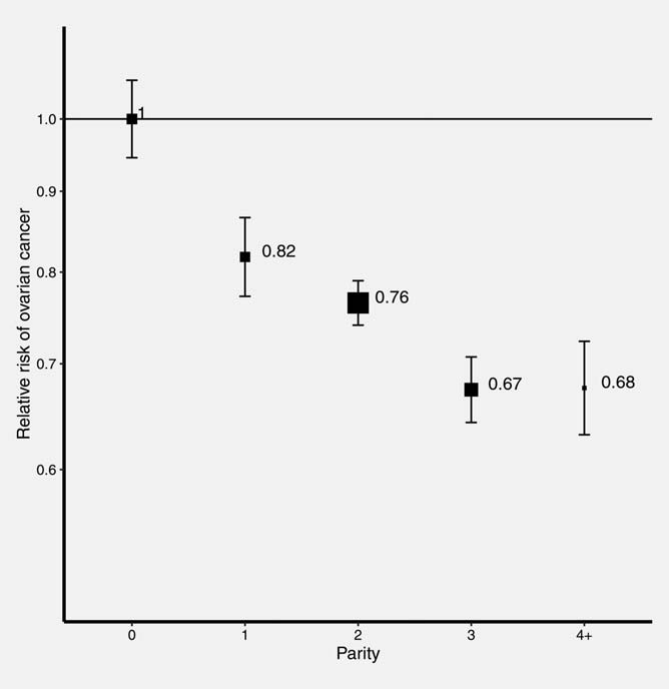
Relative risk of ovarian cancer in parous versus nulliparous women with increasing parity. Analyses are adjusted for age, region, tubal ligation, hysterectomy, family history of breast cancer, use of the oral contraceptive pill or menopausal hormones, body mass index, smoking, and socioeconomic status. The figure shows point estimates and 95% group‐specific confidence intervals.

The reduction in risk of ovarian cancer was greatest for the first birth, with almost a 20% reduction in risk compared to nulliparous women (RR = 0.82, 95% CI: 0.76–0.89); there were further reductions in risk with subsequent births, but of smaller magnitude (<10% per birth) (see Fig. 1).

**Figure 2:**
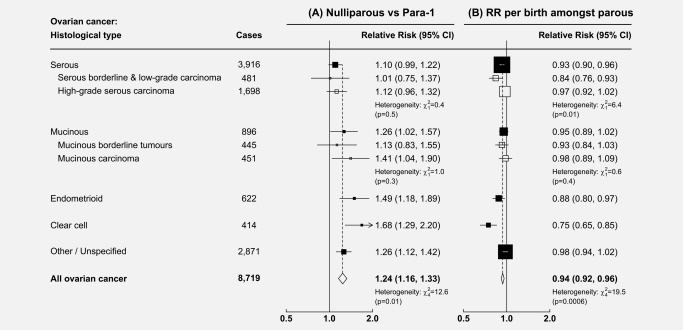
Relative risk of histological types of ovarian cancer with parity: (a) Nulliparous vs. Para‐1, (b) RR per birth amongst parous. Analyses are adjusted for age, region, tubal ligation, hysterectomy, family history of breast cancer, use of the oral contraceptive pill or menopausal hormones, body mass index, smoking, and socioeconomic status. Note: The numbers of grade‐specific serous tumours do not sum to the total number of serous tumours, as information on tumour grade was missing for 1,737 serous carcinomas.

Given the substantial difference in the proportional reduction in risk associated with the first compared to subsequent births, it was not valid to model the effects of parity simply as a relative risk per birth including nulliparous women. Instead, a model was used that allowed for distinct relative risks to be estimated for nulliparous women compared to para‐1 women, and for additional births amongst parous women. In this model, nulliparous women had a 24% higher risk of ovarian cancer than women with one child (RR = 1.24, 95% CI: 1.16–1.33), and there was significant heterogeneity by histotype (heterogeneity: p = 0.01). Nulliparous women had about a 50% higher risk of endometrioid (RR = 1.49, 95% CI: 1.18–1.89, *p* = 0.001) and almost a 70% higher risk of clear cell tumours (RR = 1.68, 95% CI: 1.29–2.20, *p* < 0.001), with a smaller increase in risk of mucinous tumours (RR = 1.26, 95% CI: 1.02–1.57, *p* = 0.03), and a small and non‐significant increase in risk of serous tumours, the most common histotype (RR = 1.10, 95% CI: 0.99–1.22) (see Fig. 2 *a*). There was no significant heterogeneity in the association between low‐grade and high‐grade serous tumours (heterogeneity: *p* = 0.5), or between mucinous borderline tumours and mucinous carcinoma (heterogeneity: *p* = 0.3).

Among parous women, each additional birth was associated with a 6% reduction in risk of ovarian cancer on average (RR = 0.94, 95% CI: 0.92–0.96). This association was heterogeneous by histotype (heterogeneity: *p* = 0.0006), with a 25% reduction in risk per birth of clear cell tumours, (RR = 0.75, 95% CI: 0.65–0.85, *p* < 0.001), a lesser reduction in the risk of endometrioid tumours (RR = 0.88, 95% CI: 0.80–0.97, *p* = 0.009), a relatively small reduction in the risk of serous tumours (RR = 0.93, 95% CI: 0.90–0.96, *p* < 0.001), and no significant reduction in risk of mucinous tumours (RR = 0.95, 95% CI: 0.89–1.02, *p* = 0.2) (see Fig. 2 *b*). When serous tumours were divided, there was a significant reduction in risk of serous borderline and low‐grade carcinoma (RR = 0.84, 95% CI: 0.76–0.93), but not of high‐grade serous carcinoma (RR = 0.97, 95% CI: 0.92–1.02) (heterogeneity: *p* = 0.01). There was no significant heterogeneity in the association between mucinous borderline tumours and mucinous carcinoma (heterogeneity: *p* = 0.4).

**Figure 3:**
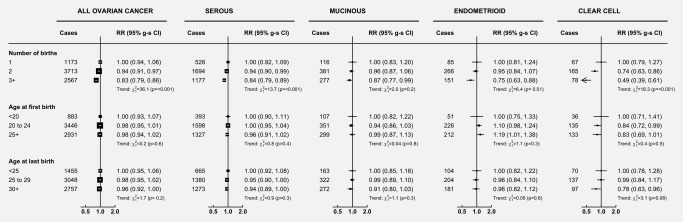
Relative risk of ovarian cancer in relation to the number of births and age at first or last birth, overall and by histotype (amongst parous women only). Analyses are adjusted for age, region, tubal ligation, hysterectomy, family history of breast cancer, use of the oral contraceptive pill or menopausal hormones, body mass index, smoking, and socioeconomic status. Analyses of the age at first and last birth are additionally stratified by parity (1, 2, 3+). The figure shows point estimates and 95% group‐specific confidence intervals.

The 24% increase in the risk of ovarian cancer amongst nulliparous women compared to women with one child was significantly greater than would have been expected based on the 6% reduction in risk per birth found amongst parous women (heterogeneity: *p* = 0.0003). There was no significant trend seen with increasing age at first or last birth, either for ovarian cancer overall, or for the four main histotypes (see Fig. 3).

### Breastfeeding and risk of ovarian cancer

**Figure 4:**
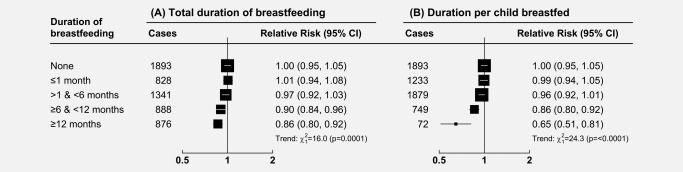
Relative risk of ovarian cancer in relation to duration of breastfeeding (amongst parous women only): (a) Total duration of breastfeeding, (b) Duration per child breastfed. Analyses are adjusted for age, region, tubal ligation, hysterectomy, family history of breast cancer, use of the oral contraceptive pill or menopausal hormones, body mass index, smoking, and socioeconomic status, and are additionally stratified by parity (1, 2, 3, 4, 5+). The figure shows point estimates and 95% group‐specific confidence intervals.

Amongst parous women with information on breastfeeding (*N* = 813,162), 68% (554,695) reported ever breastfeeding, with a mean duration of 8.1 (SD 9.4) months overall, or an average of 3.8 (SD 3.5) months per child breastfed. Women who reported breastfeeding (of any duration) had a small but significant reduction in the risk of ovarian cancer overall, compared to women who never breastfed (RR = 0.94, 95% CI: 0.89–1.00, *p* = 0.04). This was most evident amongst women who reported a total duration of breastfeeding of ≥6 months (RR = 0.90, 95% CI: 0.83–0.98 for duration ≥6 and <12 months; RR = 0.86, 95% CI: 0.79–0.94 for duration 12+ months) (see Fig. 4 *a*). A similar trend in reduced risk of ovarian cancer was seen with increasing duration of breastfeeding per child, although confidence intervals were wide in some of the longer duration groups (see Fig. 4 *b*).

**Figure 5:**
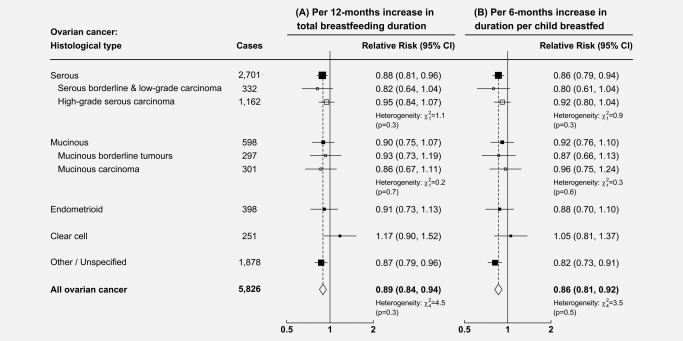
Relative risk of the main histological types of ovarian cancer in relation to duration of breastfeeding amongst parous women: (a) Per 12‐months increase in total duration for all children, (b) Per 6‐months increase in duration per child breastfed. Analyses are adjusted for age, region, tubal ligation, hysterectomy, family history of breast cancer, use of the oral contraceptive pill or menopausal hormones, body mass index, smoking, and socioeconomic status, and are additionally stratified by parity (1, 2, 3, 4, 5+). Note: The numbers of grade‐specific serous tumours do not sum to the total number of serous tumours, as information on tumour grade was missing for 1,207 serous carcinomas.

Overall, there was about a 10% reduction in the relative risk of ovarian cancer per 12‐month increase in total duration of breastfeeding (RR = 0.89, 95% CI: 0.84–0.94, *p* < 0.001), and a 14% reduction in the relative risk of ovarian cancer per 6‐month increase in duration of breastfeeding per child (RR = 0.86, 95% CI: 0.81–0.92), with no significant heterogeneity by histotype (heterogeneity: *p* = 0.3 and *p* = 0.5, respectively), but statistical power was limited (see Fig. 5).

## Discussion

We have shown, using data from a large prospective study with almost 9000 incident cases of ovarian cancer, that there is strong evidence of a reduced risk of ovarian cancer amongst parous women, with further reductions in risk with additional births, in agreement with findings from other studies.

However, there was a 24% excess overall risk of ovarian cancer amongst nulliparous women compared to women with just one child, which was substantially greater than the overall 6% reduction in risk seen with each birth in parous women. Such a difference is evident in some previously published results,^15^, ^29^ but was not formally examined before. There were differences by histotype, with about a 50–70% increase for endometrioid and clear cell tumours in nulliparous women compared to women with one child, a smaller increase for mucinous tumours, but no association with high‐grade serous tumours (the most common histotype).

The reduction in risk of ovarian cancer with parity among parous women varied significantly between the histological types. The greatest difference was seen for clear cell tumours: each additional birth amongst parous women was associated with about a 25% reduction in risk of clear cell tumours, and a weaker 10% reduction for endometrioid tumours, but was not significantly associated with the risk of high‐grade serous tumours or mucinous tumours.

Results from previous studies and pooled analyses are broadly consistent with the greater reductions in risk of endometrioid and clear cell tumours associated with childbearing,^6^, ^17^^,^ 28, 29 though individual studies did not separate nulliparous women from parous and generally had too few cases of these histotypes to show significant heterogeneity.^2^, ^5^^,^ 13, 14 ^,^ 16, 43 Our finding of no significant reduction in risk of high‐grade serous tumours (the most common histotype) with either the first birth, or on average with subsequent births, is consistent with previous findings of only a modest reduction in risk, which was statistically significant only at higher parity (para‐3 or higher).^29^

We found no significant trend in the association between parity and ovarian cancer by the age at first or last birth, which is in keeping with some studies,^4^, ^9^^,^ 16, 17 ^,^ 44, 45, 46 though not all studies.^1^, ^2^^,^ 6, 10 ^,^ 14 ^,^ 47, 48, 49, 50 The reason for different findings from different studies is unclear, but may relate at least partly to statistical power.

We found an overall reduced risk of ovarian cancer associated with longer durations of breastfeeding, with about a 10% reduction in risk of ovarian cancer per year of breastfeeding, and no significant heterogeneity of this association by histological type. A collaboration of other prospective studies has previously found no significant association between breastfeeding duration and ovarian cancer risk overall, or for the four main histotypes.^29^ The reason for the difference in findings is not clear, but might relate to differences in study size and average durations of breastfeeding.

This study is one of the largest to date, allowing for more reliable comparisons of reproductive risk factors for ovarian cancer by histological type than other studies, although power was limited even here. Strengths include its prospective design, which helped minimize differential recall of exposure history amongst women with and without cancer (a potential weakness of studies with retrospective assessment of exposures), and the virtually complete follow‐up and ascertainment of incident ovarian cancer, with only 1% of the cohort being lost to follow‐up over 14 years. A weakness is the relatively short durations of breastfeeding seen in our population: among those who reported breastfeeding, the median average duration was 3 months per child breastfed (mean: 3.8 months), and details of whether breastfeeding was exclusive to other forms of intake for the child were lacking. Another potential weakness was the lack of central histopathology review. However, as any potential misclassification of tumour histotypes would tend to blur differences, the observed variation in risk by ovarian cancer histotype is unlikely to be due to misclassification, and may even be under‐estimated here.

The substantially greater risk of endometrioid and clear cell tumours seen in nulliparous women might be at least partly related to infertility. Certain conditions may both reduce a woman's fertility and increase her risk of specific histotypes of ovarian cancer. Endometriosis may be an example of one such condition, as it reduces fertility,^51^ and pooled analyses of both retrospective^52^ and prospective^29^ studies found that women with self‐reported endometriosis had a significantly increased risk of clear cell and endometrioid cancers.

We do not have information on which of our participants were nulliparous because of infertility/subfertility, as opposed to personal choice or social circumstances. However, several other studies have shown that infertility is significantly more common amongst nulliparous women than amongst parous women.^20^, ^21^ Thus, it is probable that a substantial proportion of the nulliparous women in our study suffered from reduced fertility. Unfortunately, we do not have information on whether our participants suffered from endometriosis, or one of the many other potential causes of infertility. We thus were unable to explore what associations there may be between these specific underlying conditions and the risk of ovarian cancer.

There remains considerable uncertainty about the mechanism for the reduced risk of ovarian cancer seen with parity and breastfeeding, assuming it is causal—other than possible confounding by causes of infertility or subfertility. Popular hypotheses include the possibilities that pregnancy and lactation act by interrupting the pro‐inflammatory environment of incessant ovulation,^53^, ^54^ by modifying the hormonal environment,^55^ or by clearing pre‐malignant cells from the ovary.^1^ Our analyses do not provide support either for or against these hypotheses, although the difference in magnitude of the risk reduction between the first versus subsequent pregnancies suggests that the mechanism of risk reduction is not simply a function of time (e.g. not simply the effect of stopping ovulation for nine months of pregnancy). Whatever the underlying mechanism, it would have to account for the highly heterogeneous association by histotype found here.

In this largest prospective study of ovarian cancer to date, we found that the known reduced overall risk of ovarian cancer with increasing parity varies in magnitude between the first and subsequent births, and varies between different ovarian cancer histotypes. The findings are consistent with hypotheses that the different histotypes have different aetiologies, and that there are different effects of infertility and of births on ovarian cancer risk.

## References

[1] Adami HO , Hsieh CC , Lambe M , *et al* Parity, age at first childbirth, and risk of ovarian cancer. Lancet. 1994;344:1250–4. 7967985 10.1016/s0140-6736(94)90749-8

[2] Albrektsen G , Heuch I , Kvale G. Reproductive factors and incidence of epithelial ovarian cancer: a Norwegian prospective study. Cancer Causes Control. 1996;7:421–7. 8813430 10.1007/BF00052668

[3] Booth M , Beral V , Smith P. Risk factors for ovarian cancer: a case‐control study. Br J Cancer. 1989;60:592–8. 2679848 10.1038/bjc.1989.320 PMC2247100

[4] Braem MG , Onland‐Moret NC , van den Brandt PA , *et al* Reproductive and hormonal factors in association with ovarian cancer in the Netherlands cohort study. Am J Epidemiol. 2010;172:1181–9. 20861144 10.1093/aje/kwq264 PMC2970782

[5] Gates MA , Rosner BA , Hecht JL , *et al* Risk factors for epithelial ovarian cancer by histologic subtype. Am J Epidemiol. 2010;171:45–53. 19910378 10.1093/aje/kwp314 PMC2796984

[6] Kurian AW , Balise RR , McGuire V , *et al* Histologic types of epithelial ovarian cancer: have they different risk factors?. Gynecol Oncol. 2005;96:520–30. 15661246 10.1016/j.ygyno.2004.10.037

[7] Modugno F , Ness RB , Wheeler JE. Reproductive risk factors for epithelial ovarian cancer according to histologic type and invasiveness. Ann Epidemiol. 2001;11:568–74. 11709277 10.1016/s1047-2797(01)00213-7

[8] Moorman PG , Palmieri RT , Akushevich L , *et al* Ovarian cancer risk factors in African‐American and white women. Am J Epidemiol. 2009;170:598–606. 19605513 10.1093/aje/kwp176 PMC2732987

[9] Negri E , Franceschi S , Tzonou A , *et al* Pooled analysis of 3 European case‐control studies: I. Reproductive factors and risk of epithelial ovarian cancer. Int J Cancer. 1991;49:50–6. 1874569 10.1002/ijc.2910490110

[10] Pike MC , Pearce CL , Peters R , *et al* Hormonal factors and the risk of invasive ovarian cancer: a population‐based case‐control study. Fertil Steril. 2004;82:186–95. 15237010 10.1016/j.fertnstert.2004.03.013

[11] Purdie D , Green A , Bain C , *et al* Reproductive and other factors and risk of epithelial ovarian cancer: an Australian case‐control study. Survey of Women's Health Study Group. Int J Cancer. 1995;62:678–84. 7558414 10.1002/ijc.2910620606

[12] Riman T , Dickman PW , Nilsson S , *et al* Risk factors for invasive epithelial ovarian cancer: results from a Swedish case‐control study. Am J Epidemiol. 2002;156:363–73. 12181107 10.1093/aje/kwf048

[13] Risch HA , Marrett LD , Jain M , *et al* Differences in risk factors for epithelial ovarian cancer by histologic type. Results of a case‐control study. Am J Epidemiol. 1996;144:363–72. 8712193 10.1093/oxfordjournals.aje.a008937

[14] Titus‐Ernstoff L , Perez K , Cramer DW , *et al* Menstrual and reproductive factors in relation to ovarian cancer risk. Br J Cancer. 2001;84:714–21. 11237375 10.1054/bjoc.2000.1596 PMC2363792

[15] Tsilidis KK , Allen NE , Key TJ , *et al* Oral contraceptive use and reproductive factors and risk of ovarian cancer in the European Prospective Investigation into Cancer and Nutrition. Br J Cancer. 2011;105:1436–42. 21915124 10.1038/bjc.2011.371 PMC3241548

[16] Tung KH , Goodman MT , Wu AH , *et al* Reproductive factors and epithelial ovarian cancer risk by histologic type: a multiethnic case‐control study. Am J Epidemiol. 2003;158:629–38. 14507598 10.1093/aje/kwg177

[17] Yang HP , Trabert B , Murphy MA , *et al* Ovarian cancer risk factors by histologic subtypes in the NIH‐AARP Diet and Health Study. Int J Cancer. 2012;131:938–48. 21960414 10.1002/ijc.26469 PMC3505848

[18] Ness RB , Cramer DW , Goodman MT , *et al* Infertility, fertility drugs, and ovarian cancer: a pooled analysis of case‐control studies. Am J Epidemiol. 2002;155:217–24. 11821246 10.1093/aje/155.3.217

[19] Tworoger SS , Fairfield KM , Colditz GA , *et al* Association of oral contraceptive use, other contraceptive methods, and infertility with ovarian cancer risk. Am J Epidemiol. 2007;166:894–901. 17656616 10.1093/aje/kwm157

[20] Stephen EH , Chandra A. Declining estimates of infertility in the United States: 1982–2002. Fertil Steril. 2006;86:516–23. 16952500 10.1016/j.fertnstert.2006.02.129

[21] Bushnik T , Cook JL , Yuzpe AA , *et al* Estimating the prevalence of infertility in Canada. Hum Reprod. 2012;27:738–46. 22258658 10.1093/humrep/der465 PMC3279129

[22] Luan NN , Wu QJ , Gong TT , *et al* Breastfeeding and ovarian cancer risk: a meta‐analysis of epidemiologic studies. Am J Clin Nutr. 2013;98:1020–31. 23966430 10.3945/ajcn.113.062794 PMC3778857

[23] Prat J. Pathology of cancers of the female genital tract. Int J Gynaecol Obstet. 2015;131 Suppl 2:S132–45. 26433670 10.1016/j.ijgo.2015.06.010

[24] Kurman RJ , Shih IM. Molecular pathogenesis and extraovarian origin of epithelial ovarian cancer–shifting the paradigm. Hum Pathol. 2011;42:918–31. 21683865 10.1016/j.humpath.2011.03.003 PMC3148026

[25] Collaborative Group On Epidemiological Studies Of Ovarian Cancer . Ovarian cancer and oral contraceptives: collaborative reanalysis of data from 45 epidemiological studies including 23,257 women with ovarian cancer and 87,303 controls. Lancet. 2008;371:303–14. 18294997 10.1016/S0140-6736(08)60167-1

[26] Collaborative Group On Epidemiological Studies Of Ovarian Cancer . Menopausal hormone use and ovarian cancer risk: individual participant meta‐analysis of 52 epidemiological studies. Lancet. 2015;385:1835–42. 25684585 10.1016/S0140-6736(14)61687-1 PMC4427760

[27] Collaborative Group On Epidemiological Studies Of Ovarian Cancer . Ovarian cancer and smoking: individual participant meta‐analysis including 28,114 women with ovarian cancer from 51 epidemiological studies. PLoS Med. 2012;13:946–56. 10.1016/S1470-2045(12)70322-4 PMC3431503 22863523

[28] Pearce CL , Rossing MA , Lee AW , *et al* Combined and interactive effects of environmental and GWAS‐identified risk factors in ovarian cancer. Cancer Epidemiol Biomarkers Prev. 2013;22:880–90. 23462924 10.1158/1055-9965.EPI-12-1030-T PMC3963289

[29] Wentzensen N , Poole EM , Trabert B , *et al* Ovarian Cancer Risk Factors by Histologic Subtype: An Analysis From the Ovarian Cancer Cohort Consortium. J Clin Onc. 2016;34:2888–98. 10.1200/JCO.2016.66.8178 PMC5012665 27325851

[30] The Million Women Study Collaborative Group . The Million Women Study: design and characteristics of the study population. The Million Women Study Collaborative Group. Breast Cancer Res. 1999;1:73–80. 11056681 10.1186/bcr16 PMC13913

[31] World Health Organization . International Statistical Classification of Diseases and Related Health Problems., 10th ed. Geneva: World Health Organization, 1992.

[32] Percy C , Van Holten V , Muir CS. International Classification of Diseases for Oncology: ICD‐O., 2nd ed. Geneva: World Health Organization, 1990.

[33] Fritz A , Percy C , Jack A , *et al* International Classification of Diseases for Oncology: ICD‐O., 3rd ed. Geneva: World Health Organization, 2000.

[34] Gaitskell K , Green J , Pirie K , on behalf of the Million Women Study Collaborators , *et al* Tubal ligation and ovarian cancer risk in a large cohort: Substantial variation by histological type. Int J Cancer. 2016;138:1076–84. 26378908 10.1002/ijc.29856 PMC4832307

[35] Townsend P , Phillimore P , Beattie A. Health and Deprivation: inequality and the North. London: Croom Helm, 1988.

[36] Lunn M , McNeil D. Applying Cox regression to competing risks. Biometrics. 1995;51:524–32. 7662841

[37] Plummer M. Improved estimates of floating absolute risk. Stat Med. 2004;23:93–104. 14695642 10.1002/sim.1485

[38] Easton DF , Peto J , Babiker AG. Floating absolute risk: an alternative to relative risk in survival and case‐control analysis avoiding an arbitrary reference group. Stat Med. 1991;10:1025–35. 1652152 10.1002/sim.4780100703

[39] StataCorp . Stata Statistical Software: Release 14. College Station, TX: StataCorp LP, 2015.

[40] R Development Core Team . R: A Language and Environment for Statistical Computing. Vienna, Austria: R Foundation for Statistical Computing, 2015.

[41] Wickham H. ggplot2: Elegant Graphics for Data Analysis. : Springer‐Verlag New York, 2009.

[42] Arnold M. Jasper: Jasper makes plots. R package version 2–192., 2015.

[43] Fortner RT , Ose J , Merritt MA , *et al* Reproductive and hormone‐related risk factors for epithelial ovarian cancer by histologic pathways, invasiveness and histologic subtypes: Results from the EPIC cohort. Int J Cancer. 2015;137:1196–208. 25656413 10.1002/ijc.29471 PMC6284794

[44] Chiaffarino F , Pelucchi C , Parazzini F , *et al* Reproductive and hormonal factors and ovarian cancer. Ann Oncol. 2001;12:337–41. 11332145 10.1023/a:1011128408146

[45] Cramer DW , Hutchison GB , Welch WR , *et al* Determinants of ovarian cancer risk. I. Reproductive experiences and family history. J Natl Cancer Inst. 1983;71:711–6. 6578366

[46] Vachon CM , Mink PJ , Janney CA , *et al* Association of parity and ovarian cancer risk by family history of breast or ovarian cancer in a population‐based study of postmenopausal women. Epidemiology. 2002;13:66–71. 11805588 10.1097/00001648-200201000-00011

[47] Bevier M , Sundquist J , Hemminki K. Does the time interval between first and last birth influence the risk of endometrial and ovarian cancer?. Eur J Cancer. 2011;47:586–91. 21055917 10.1016/j.ejca.2010.10.004

[48] Cooper GS , Schildkraut JM , Whittemore AS , *et al* Pregnancy recency and risk of ovarian cancer. Cancer Causes Control. 1999;10:397–402. 10530609 10.1023/a:1008960520316

[49] Whiteman DC , Siskind V , Purdie DM , *et al* Timing of pregnancy and the risk of epithelial ovarian cancer. Cancer Epidemiol Biomarkers Prev. 2003;12:42–6. 12540502

[50] Yang CY , Kuo HW , Chiu HF. Age at first birth, parity, and risk of death from ovarian cancer in Taiwan: a country of low incidence of ovarian cancer. Int J Gynecol Cancer. 2007;17:32–6. 17291228 10.1111/j.1525-1438.2007.00804.x

[51] Hickey M , Ballard K , Farquhar C. Endometriosis. BMJ. 2014;348:g1752 24647161 10.1136/bmj.g1752

[52] Pearce CL , Templeman C , Rossing MA , *et al* Association between endometriosis and risk of histological subtypes of ovarian cancer: a pooled analysis of case‐control studies. Lancet Oncol. 2012;13:385–94. 22361336 10.1016/S1470-2045(11)70404-1 PMC3664011

[53] Fathalla MF. Incessant ovulation and ovarian cancer ‐ a hypothesis re‐visited. Facts Views Vis Obgyn. 2013;5:292–7. 24753957 PMC3987381

[54] Fathalla MF. Incessant ovulation–a factor in ovarian neoplasia?. Lancet. 1971;2:163 4104488 10.1016/s0140-6736(71)92335-x

[55] Risch HA. Hormonal etiology of epithelial ovarian cancer, with a hypothesis concerning the role of androgens and progesterone. J Natl Cancer Inst. 1998;90:1774–86. 9839517 10.1093/jnci/90.23.1774

